# PSP is a promising biomarker of sepsis; however, potential elimination by RRT must be considered

**DOI:** 10.1186/s13054-022-04214-7

**Published:** 2022-11-07

**Authors:** Patrick M. Honore, Sebastien Redant, Pharan Djimafo, Sydney Blackman, Ibrahim Bousbiat, Emily Perriens, Thierry Preseau, Bogdan Vasile Cismas, Keitiane Kaefer, Leonel Barreto Gutierrez, Sami Anane, Andrea Gallerani, Rachid Attou

**Affiliations:** 1 ICU Department, Centre Hospitalier Universitaire Brugmann, Brugmann University Hospital, Place Van Gehuchtenplein, 4, 1020 Brussels, Belgium; 2 Centre Hospitalier Universitaire Brugmann, ULB University, Brussels, Belgium; 3 ED Department, Centre Hospitalier Universitaire Brugmann, Brussels, Belgium

According to Lopez et al. pancreatic stone protein (PSP) is “a promising biomarker to diagnose infections in hospitalized patients,” distinguishing infections from non-infections and outperforming C-reactive protein (CRP) and procalcitonin (PCT) [1]. PSP is a 16 kDa C-type lectin protein [1]. In patients admitted for sepsis, the value of PSP at admission correlates with the Sequential Organ Failure Assessment (SOFA) score and can predict intensive care unit (ICU) mortality [1]. Lopez et al. based most of their findings on a study looking at 90 severe patients in a burn ICU [2]. Septic shock induced acute kidney injury (AKI) in at least 50% and 25% needed continuous renal replacement therapy (CRRT) [3]. However, looking at the molecular weight of PSP, we do need to consider potential elimination by RRT using membranes with a cut off of 35–40 kDa [4]. New highly adsorptive membranes (HAM) could also absorb PSP in addition to the removal by convection [5]. Since the risk of elimination by CRRT is very likely, PSP is potentially unreliable for predicting either septic shock or patients needing to be transferred to the ICU. Before analyzing the accuracy of PSP studies are needed to determine if PSP is significantly eliminated or not by CRRT.

## Data Availability

Not applicable.

## Abbreviations

PSP: Pancreatic stone protein
CRP: C reactive protein
PCT: Procalcitonin
SOFA: Sequential organ failure assessment
ICU: Intensive care unit
AKI: Acute kidney injury
CRRT: Continuous renal replacement therapy
HAM: High adsorptive membranes
